# Book Reviews

**Published:** 1916-11

**Authors:** 


### BOOK REVIEWS

---

Books mentioned may be inspected at and ordered through this office. So far as possible, books received in any month will be reviewed in the issue of the second month following.

---

**Universal Military Education and Service:** The Swiss System for the U. S. Lucien Howe, M. D., Buffalo. Fellow of the Royal Society of Medicine, Member of the Royal College of Surgeons, Professor Emeritus of Ophthalmology, University of Buffalo. G. P. Putnam's Sons, N. Y. 138 pages, $1.00.

This work by our distingushed fellow townsman is not merely an advocacy of military training for its physical and disciplinary value and as an insurance of peace, but a collection of definite facts bearing on the subject. It is arranged in the form of questions and answers. The literary style is pleasing, in spite of the effort at condensation and the attitude of the author so conservative as to carry conviction. We have often said that physicians ought to be selected to prepare works on law and science, as well as on sociologic questions, from their professional habit of dealing with facts, getting at the ultimate truth, and sense of obligation to search authorities for available sources of information. In these respects, this book by a physician, is far ahead, chapter by chapter, of any discussion of military preparedness by military men that we have encountered, so far as the education of the people is concerned. It is estimated that the annual expense for military preparedness, $750,000,000 a year, is insurance at the rate of ½% per year—about the same as for insurance against fire. As we spend 2 billion a year for liquors, 1 billion for tobacco, 800 million for jewelry and plate, 85 million for millinery and 80 million for patent medicines, the question as to whether we can afford this insurance is answered at a glance. Chewing gum alone, would cover the expense of two battle ships a year. The argument that military training incites to violence is answered by the statistics of murders which are, per 100,000 population per year, 0.9 in London, 2. in Berlin, 3.5 in Paris, 7.1 in N. Y., 9. in Chicago, 13.4 in San Francisco, 30 in Charleston and 68 in Memphis. The physical advantages of the Swiss and Australian system of military training in the public schools and, conversely, the educational advantages connected with the comparatively brief additional periods of actual camp service or of service in the regular army or navy, are well presented. While neither an alarmist nor a partisan, the author does not mince words in dealing with the possible dangers to which our country is exposed. Indeed, he presents a table showing the number of days and size of armies required to invade our shores from various foreign countries. The book is one which every citizen ought to read carefully.

---

**Disorders of the Sexual Function in the Male and Female.** Max Huhner, M. D., N. Y. F. A. Davis Co., Philadelphia. 318 pages, $3.00.

This is a practical discussion free, on the one hand, from prolixity and the undue relation of case histories, on the other hand from false modesty or hypocrisy. The author shows a practical familiarity with the subject, obtained from extensive experience.

---

**The Christian Church.** Wm. McAfee Goodwin, L. B., C. S., Washington, author and publisher. 165 pages including index, $1.50.

This book is not directly intended as a means of proselyting but, on the contrary is "a timely, impersonal, dispassionate, analytical, unanswerable discussion of the weaknesses and inconsistencies of the Christian Science Church organiztion." For example, the author holds that the refusal of that organization to receive or instruct one who has been a member of the Roman Catholic Church, is inconsistent with the general policies of all churches. None of the chapters deal directly with the conflict between the medical profession and Christian Scientists. A table states the number of C. S. mind-healing practitioners at 5,616, of whom 4,986 are women. 5,090 of the total and 4,521 of the women are in the U. S. It is stated that the general rules of the C. S. Church require obedience to the civil law first, even if contrary to the regulations of the Church.

---

**Jubilee Souvenir**—1866-1916, Parke, Davis & Co.

Beginning with the drug stores of Dr. Samuel P. Duffield in Detroit, gradually supplemented with a small manufacturing plant for Gålenicals, Hervey C. Parke joining the firm in 1866 and George S. Davis in 1867, the present large plant with its numerous American and foreign branches has gradually developed. Other early leaders were Theodore D. Buhl, Wm. M. Warren and H. A. Wetzel. The present directors are: Frank G. Ryan, president; David C. Whitney and Henry M. Campbell, vice-presidents; E. G. Swift, secretary and general manager; Jas. E. Bartlett, assistant secretary and treasurer; George Hargreaves, treasurer; N. H. F. McLeod, auditor, and Arthur H. Buhl, Chas. Stinchfeld, directors. Among the new drugs presented to the profession by this house are: viburnum, prunifolium, guarana, eucalyptus, coca, jaborandi, Janaica dogwood, saw palmetto, convallaria, pichi, cocillana, kamala and areca nuts. From a beginning with Galenicals and new crude drugs and available pharmacologic preparations, research work early established and gradually extended along new lines brought out the necessity of preparing active principles and for the physiologic standardization of Galenicals, and later of biologic products. Illustrations of the various branch establishments and a few of the main plant are given. Very few business firms or indeed institutions of any kind can look back on so long and successful a period of growth.

